# Application of Close Range Photogrammetry to Deck Measurement in Recreational Ships

**DOI:** 10.3390/s90906991

**Published:** 2009-09-03

**Authors:** Celestino Ordóñez, Belén Riveiro, Pedro Arias, Julia Armesto

**Affiliations:** Departamento de Ingeniería de los Recursos Naturales y Medio Ambiente, Universidad de Vigo, E.T.S.I. Minas, Rúa Maxwell s/n, 36310 Vigo, Spain; E-Mails: belenriveiro@uvigo.es (B.R.); parias@uvigo.es (P.A.); julia@uvigo.es (J.A.)

**Keywords:** close range photogrammetry, coordinate measurement machine, 3D model, teakwood

## Abstract

In this article, we present results that demonstrate the utility of close range photogrammetry in the measurement of decks in recreational craft as an alternate measurement system to the one based on direct acquisition of coordinates. The areas of deck covered with teakwood for aesthetic or security reasons were measured. Both methods were compared in terms of precision of measurements, time consumption, equipment cost, and ease of manipulation and equipment transportation. Based on the results, we conclude that photogrammetry has advantages in almost every aspect with respect to the direct method. Consequently, photogrammetry is suggested as a suitable method for coordinate measurement of decks in recreational ships. However, in some special circumstances, where ships have wide corridors with few obstacles the direct method can be more appropriate than the photogrammetric method.

## 1. Introduction

The recreational craft industry, which includes the building of crafts, motors and equipment, as well as the associated services sector, has an important economic impact on employment and turnover in Europe [1,2]. Thirty two million Europeans enjoy the use of recreational boats each year, generating a total of 253,000 jobs in the EU; this makes it one of the sectors with the greatest potential for growth within the maritime field, with an estimated growth of 5–6% a year, as well as demonstrating its significant effect on regional employment [3,4]. The recreational craft industry is characterised by products with high added value and by the use of manufacturing techniques with fairly low technical level, where the skill and experience of workers is decisive in the quality of the final product.

In situations where in the serial automated replication of an existing boat model is needed, reverse engineering techniques [5,6] may be employed to obtain digital models of boats [7] during boat construction, The use of these techniques is justified in processes where geometric accuracy is required: for example, in the manufacture of wood decks (generally teakwood-*Tectona grandis*) and other visible elements in this kind of crafts. The measurements taken on the decks are a key factor when shapes and areas need to be calculated with accuracy in order to be covered with the mentioned material. Different techniques and equipments may be used: 3D laser scanners [8], theodolites and total stations, close range photogrammetry (CRP) [9], coordinates measurement machines (CMM) [10], and structured light systems [11]. Due to the characteristics of the measured pieces in this work and restrictions in the cost, size and weight of the equipments, the most suitable systems are CRP and low cost CMMs; thus, this paper only contains the results of a comparative survey of these two systems. In fact, we have analyzed only a specific CMM, the Proliner 5.7, since it is the equipment used by the company that has partially supported this work. This is a small company that cannot afford great expenditures on equipment, so we are only interested in low cost systems.

3D laser scanner systems provide very good results when the element being modelled has a complex shape and a considerable size [12], but it is not as effective when the object has less complexity; also, the high cost of this equipment prohibits its application in the situation analysed in the present research. Moreover, the structured light systems are competitive in situations where the modelled object is small, and high accuracy is required [13], but the cost of this technology is also quite high. In the last years, portable laser trackers are being extensively used for many industrial applications, such as shipbuilding [14]. Laser trackers accuracy and speed distinguish them from other portable coordinate measuring instruments. Because an operator can make rapid measurements with a minimum of advance preparation, trackers are among the most versatile of the coordinate measuring instruments. However, they were not tested in this work mainly because of their cost, but also because they are relatively large and heavy.

Typical land surveying equipments, such as theodolites and total stations [15], have low to medium costs, but they are not appropriate for measuring boat decks: they are labour intensive and when a few millimetres accuracy is required the cost of these systems increases substantially [16].

Although they are very accurate devices, classical bridge and arm CMMs were initially discarded since we are interested in portable systems and also due to their high cost, size and weight [17]. Portable arm CMMs were also analyzed, but they are expensive, quite large and not practical for the measurement of large objects such as the corridors of the boats.

Measuring the areas where teakwood will be located in a fast and accurate way is essential to reduce cost; because when errors are too large, the measurements must be taken again, and the templates have to be constructed and placed in the craft a second time. This causes significant economic loss, considering that workers have to move long distances from the factory to the ships, frequently through different countries.

The aim of this work is to determine the most suitable measurement method, in terms of accuracy in measurements, costs, size and weight of equipment, and ease of measurement method and data processing. In fact, our work can be considered as an experimental study rather than a systematic methodology analysis.

## 2. Materials and methods

### 2.1. Direct Measurement Equipment

**Figure 1.**
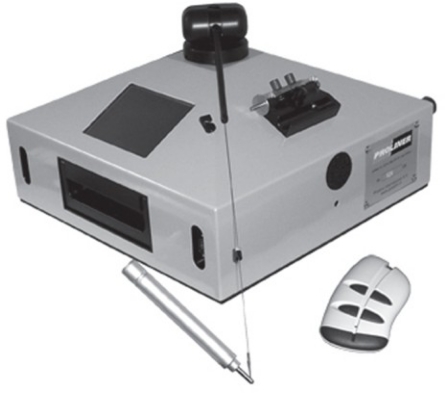
Direct measurement equipment where the central unit and pen joined by cable can be distinguished.

The CMM equipment used for direct measurement of the decks was a Proliner 5.7 manufactured by PRODIM International (Figure 1). This system is especially designed for fast and accurate measurement 2D and 3D contours. The measurement is taken by locating a contact device, similar to a pen, on each point that defines the contours of the boat deck. This pen has a spherical tip that is joined to the machine by a cable. The 3D coordinates (X, Y, Z) of each point are stored in a memory device, and the machine generates a dxf file where all the points are joined by means of straight lines. As a result, all the data can be exported to CAD systems, where all the contours of pieces are delineated. Then, they are exported to a Computer Aided Manufacturing (CAM) program that converts the CAD geometry into tool-paths. Tool-paths are the paths the tool will take around and through the piece of material to create the part. The command language for tool paths is called G-code, the common name for the programming language of computer numerical controlled (CNC) Machines. This code can be understood by the CNC machine where the wood is cut according to the dimensions of the objects represented in the CAD files.

Proliner is a robust and compact machine that is designed to work under difficult conditions. It can be manipulated by a single person, and it is also easy to use after a short period of training. In order to make the measurements easily, Proliner 5.7 can be located in different positions: horizontal, vertical and tilted.

The Proliner has a cable with a length of 5 meters, so the maximum distance that can be measured with the Proliner from a station position is 10 meters (in absence of obstacles). The technical specifications of Proliner, according to the manufacturer, are the following:

Dimensions: 420 × 420 × 250 mm
Weight: 10 kg
Rechargeable batteries with an estimated capacity of 2 h.
Temperature range: 0–40 °C
Precision in point coordinates: 0.3 mm.
Capacity of storage: 32 MB.

### 2.2. Direct Measurement Method

The first task when a craft is being measured using the Proliner as a previous study in order to estimate the best positions for the machine to be located (stations). Thus, it is possible to reduce the number of stations and, consequently, the time of measurement and the final error in the coordinates of the points, since error propagation is also reduced. When we are measuring a craft, we have two elements to determine:

- Corridors (to port and to starboard): they are normally narrow and elongated; thus, several Proliner stations are required in order to determine the whole area of the piece.
- Thwarts, cockpits, and solariums: they are shorter and wider pieces, so they can be measured with the Proliner using very few stations (normally one to four stations).

According to the manufacturer of the equipment, a minimum of three points are required to link two consecutive stations. As a result, the parameters of an affine transformation (translation and rotations) between the coordinate systems of different Proliner stations can be established. Instead, we used four tie points for the transformation in order to have redundant information, and we solved the transformation equations system by means of a least squares adjustment.

Although the company who works with the Proliner machine does not check the errors caused by the coordinate systems adjustments, we proposed, mainly in corridors, to distribute the stations around a closed traverse, so that the initial and final stations are the same. This way the closing error can be known, and, if it is acceptable according to the company requirements, it can be taken into account by means of a least-squares adjustment [18]. The general expression of the system of equations that must be solved is the following:

(1) $$\begin{array}{l} \left[R_{2}\right]\left[X_{2a}\right]+T_{2}-X_{1b}=0\;\\ \left[R_{3}\right]\left[X_{3a}\right]+T_{3}-\left[R_{2}\right]\left[X_{2b}\right]-T_{2}=0\\ ......................................................\\ \left[R_{N}\right]\left[X_{Na}\right]+T_{N}-\left[R_{N-1}\right]\left[X_{N-1b}\right]-T_{N-1}=0\\ \left[R_{N}\right]\left[X_{Nb}\right]+T_{N}-X_{1a}=0 \end{array}$$

where:

[*R_i_*]: rotation in station i
[*T_i_*]: translation in station i
[*X_ia_*]: column vector with the coordinates of the quadrilateral measured backsight from station i
[*X_ib_*]: column vector with the quadrilateral coordinates measured, foresight, from station i
*N*: number of stations

The process of reading the original Proliner dxf file, adjusting the coordinates and writing the final dxf file with the adjusted coordinates was automated by writing a C++ program.

A very important drawback in using the Proliner on corridors is that they are normally very narrow, so it is very difficult (if not impossible) to put it on the deck. Also, the cable trips over the deck, which reduces its effective reach considerably and forces more stations to measure the entire corridor (in an average corridor, it is frequent to use six or more stations). Finally, increasing the number of stations entails a longer measurement time and greater error in the coordinates of the points that define the corridor. Therefore, it is important to invest the necessary time to analyse the best locations for the stations before beginning the measurement. When it is not possible to use the Proliner, a solution is to put a plastic material over the deck and cut it following the contour of the corridor. Of course, this is a extremely slow procedure.

Files provided by the Proliner are imported by a CNC machine where wood templates are constructed. These templates are placed on the boat and adjusted to fit adequately. Finally, the adjusted templates are used as a pattern to construct the teak boards that will cover the deck.

### 2.3. Indirect Measurement Equipment

The measurements by close range photogrammetry were performed with a digital camera Nikon D200. The technical specifications are:

- Charge-coupled device (CCD) image sensor with 12 Megapixels
- Format: 23.7 × 15.70 mm (3872 × 2595 pixels)
- Weight: 830 g
- Storage in flash memory card
- LCD monitor 2.5 in., which allows images to be visualised
- USB and video connections
- Remote control options

The lens used for the measurement is a wide angle Nikkor 20 mm f/2.8D, digital angle with a view of 70 degrees. In order to build a 3D model of an object by photogrammetry, it is necessary to solve the following steps: interior and exterior orientation [19]. The interior orientation consists of reconstructing the perspective rays in the camera, using the values obtained in the calibration process: radial and decentering lens distortion, focal length and position of the principal point. By means of interior orientation we can get rid of errors that arise from the use of non-metric cameras. The exterior orientation consists of identifying the position of the cameras when the images were impressed on the sensor.

The camera calibration was performed by means of a self-calibration bundle adjustment method by taking shots on a calibration grid [19,20]. The following are the interior orientation parameters of the camera:

Focal distance: 20.7836 mm ± 0.008 mm
Principal point: (11.9327, 8.2262) mm
Radial distortion parameters:
  $$\begin{array}{l} K_{1}=2.687\times 10^{-4}{\text{mm}}^{-1}\pm 4.1\times 10^{-7}\\ K_{2}=-4.191\times 10^{-7}{\text{mm}}^{-1}\pm 2.1\times 10^{-9} \end{array}$$
Tangential distortion parameters: negligible
Decentering distortion parameters:
  $$\begin{array}{l} P_{1}=-7.104\times 10^{-6}{\text{mm}}^{-1}\pm 6.9\times 10^{-7}\\ P_{2}=-2.040\times 10^{-6}{\text{mm}}^{-1}\pm 7.6\times 10^{-7} \end{array}$$

**Figure 2.**
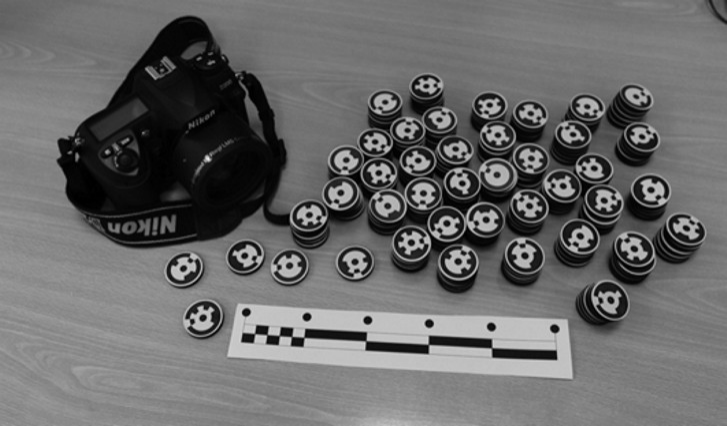
Indirect measurement equipment: digital camera, coded targets and scale bar.

The photogrammetric equipment is completed with coded targets of type RAD (ringed automaticlally detected target) [21,22] in black and white, which are located on the object surface that is being modelled, and scalar bars, which allow the photogrammetric models to be scaled (Figure 2).

### 2.4. Indirect Measurement Method

#### Data acquisition

Data acquisition was carried out according to monoscopic photogrammetry principles in order to ensure better geometry between homologous rays and to improve measurement accuracy [23]. The shots were taken considering the following points:

1. Each element depicted must be contained in a minimum of three photographs.
2. The convergence between photographs taken from different positions should have optimum values between 60° and 90° so that the beam adjustments will be carried out well.
3. There must be at least 50% overlap between consecutive photographs.

In order to have accurate measurements, the sensitivity of the CCD sensor should be adapted to the illumination conditions. Also, in many cases, it is necessary to activate the flash, especially when photographs are being taken in the shipyard plants.

The distance between the camera and the object is approximately of 1.5 meters (for a man of average height), so many photographs are necessary in order to cover all the ship contours. It is recommended that more photographs than the minimum be taken to reduce the need to return to the ship to take photos again due to problems in the first set (bad exposure, blurred photos, or insufficient overlap) The quantity of photographs needed is larger when a flash is required, because the ends of the photographs are dark, and consequently, the effective model surface is smaller.

**Figure 3.**
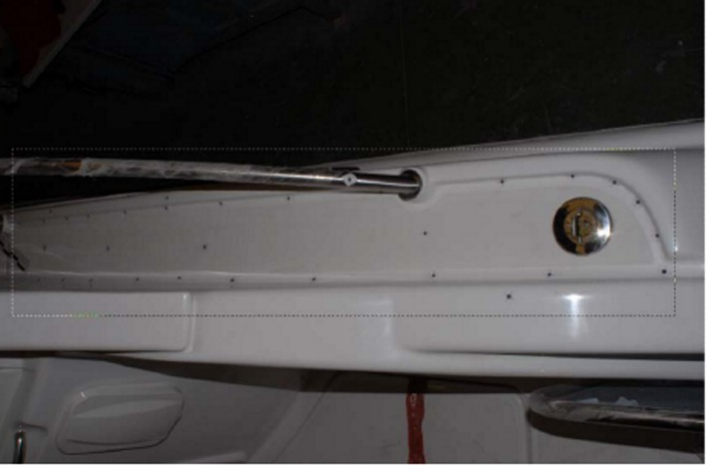
Targets located on straight and curved lines on a corridor.

Due to the white colour and rounded forms of the deck contours, they cannot be distinguished clearly in the photographs. Thus, it is necessary to mark them by using targets located in points that define the shape of the contours (Figure 3).

In order to scale the photogrammetric model, scale bars whose dimensions are known are located in the zone that is being measured. The bars have different sizes, and their total length varies between 30 and 50 cm. These dimensions allow them to be placed into a case with the rest of the equipment.

#### Data processing

Once the data acquisition is completed, the photographs are downloaded to the PC, and the photogrammetric process starts with the interior orientation, where the perspective rays of the camera are reconstructed. After that, external orientation is performed in two steps: relative and absolute orientation. The evaluation of the exterior orientation elements of one camera with regard to the photo coordinate system of another is known as relative orientation. The simultaneous intersection of at least five pairs of homologue rays distributed through the model is adequate for the remaining points to intersect as well, according to perspective geometry. The rays’ equations are calculated analytically, and the relative orientation parameters can be calculated by applying the collinearity (coplanarity) conditions to the homologous rays (vectors defining the projection of every ground point on the photographs) for each pair of photos [24].

The link points are defined by means of coded targets as those used for contour definition. Once the model has been established, we must adjust it to the ground coordinate system by means of absolute orientation. For our particular case, absolute orientation consists of scaling the 3D model and then levelling it by defining a horizontal plane with three points obtained from the images. The scale factor is attained by measuring the real distances between the points marked in the scale bars and comparing them with the distances in the model.

Once the models were joined and scaled, the coordinates of the points were defined by the targets. Then, the contours of the craft’s elements can be drawn. Points can be joined by means of straight or curved lines (for example, b-splines [25]) in order to obtain smoother shapes. When we work with coded targets [26], the identification of the centre of each one is completed automatically; thus, time consumption is reduced (approximately 80%). If coded targets are not used in these tasks, the time consumption of the point restitution is unacceptable for the company. The 3D models can be exported to conventional CAD software and numeric control machines (dxf, dwg, dxb, dgn, etc.).

## 3. Results and Discussion

Before beginning the deck measurements, the Proliner was calibrated by the manufacturer, and several tests were performed in the laboratory to obtain the relative accuracy of both measurement systems. Measurements were performed on points located in a wooden grid built by a numeric control machine. The measurements taken with the Proliner were acquired from only one position, and the photogrammetric measurements were performed with a total of four models. The average of the standard deviations between the coordinates measured by both systems was 0.56 mm in the (X,Y) coordinates and 0.92 mm in the Z coordinate.

After proving that Proliner and the camera were well calibrated, and consequently that the errors were inside the admissible margins for each piece of equipment, the deck measurement was started. Several models of ships manufactured by Rodman Polyships in a shipyard in Vigo (Spain) were measured.

**Figure 4.**
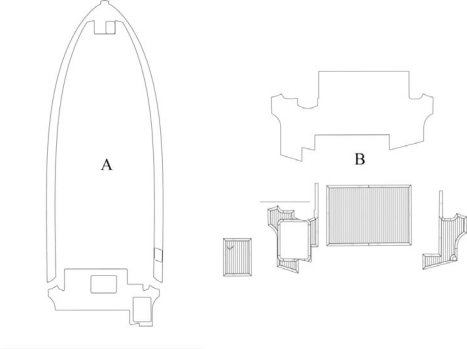
Outlined plans of corridors (A) and cockpit (B) for a Rodman 49 ship.

Figure 4 shows two contours of the different parts measured on the deck of the biggest Rodman yacht (A), and one example of an outlined cockpit, which is used to manufacture the teak pieces that will be installed on the ship deck (B).

The maximum number of different Proliner positions in the three examples for the cockpits shown in this work was three. The measurement tasks were performed accurately in order to avoid errors caused by operators (e.g., equipment movement or inadequate precision in the measurement of a point that is used to connect different Proliner positions). The maximum number of models in the photogrammetric method was 15 in cockpits and 17 in corridors. In order to facilitate the comparison between both measurement methods, the coordinates of the same points were determined.

In Table 1, the absolute and relative values of the difference between perimeters (mm) and areas (cm^2^) of the pieces with high complexity are shown, and they correspond to the measurements taken in the Rodman shipyard. Only the results for the largest corridor, Rodman 49, are presented in this work. The measurements of this corridor involved six Proliner stations, and corridors of the smaller ships were not measured because they were too narrow for the Proliner machine.

In Table 2, the time consumption of the 3D measurement of corridors and cockpits for each ship is shown, and it corresponds to field work and laboratory tasks. According to these results, photogrammetry requires more time than the direct method in data acquisition (fieldwork) for cockpits, but it is faster for corridor data acquisition. Also, photogrammetry is the only method, from the two compared, that allowed the measurement of the corridors in all the cases. Taking into account that the best methodology for decks measurement should permit the measurement of all the elements, it seems reasonable to say that the photogrammetric method is the most suitable for this type of work.

Another advantage of photogrammetry is based on the fact that all the photogrammetric equipment is lightweight and can be placed in a small case. This question is very important for the company because they often have to transport the equipment by plane. The Proliner has to be checked, while the photogrammetric equipment can be transported as hand bags. This way costs associated with misplaced baggage are avoided, and these costs are both important and not frequently budgeted. The third advantage of photogrammetry is that the photogrammetric equipment is about 85% cheaper than the Proliner.

On the other hand, the direct method has several advantages in measuring cockpits, solariums and other small elements because they can be measured using only a few stations. In these cases, data acquisition is faster and error propagation is not a significant problem. However, this advantage is not too relevant because the ideal method should be used to measure all the pieces, including corridors.

Another advantage of the direct method is that it requires less training time for fieldwork and data processing. In fact, with an application that links different stations and makes error adjustments, it is not necessary to manually link points measured from two positions in CAD software.

Photogrammetry is not recommended when the ship is located in dimly illuminated places where flashes are not enough to obtain bright photographs (but this situation is very unusual). The direct method is also advantageous in that it can be used on ships because there are not many obstacles where the Proliner cable can trip with the obstacles, but this is very unusual.

## 4. Conclusions

We have demonstrated a suitable method to obtain 3D models of decks in recreational ships. Initially, two methods for 3D measurements were considered: one based on direct acquisition of coordinates, and the other based on indirect acquisition by close range photogrammetry.

After several tests in three different recreational crafts, we concluded that, in general terms, photogrammetry has several advantages. First, photogrammetry equipment is cheaper and more versatile. Photogrammetry also has the capacity to measure all the elements that are present in the ship deck, including corridors. However, in some special circumstances, where ships have wide corridors with few obstacles the direct method can be more appropriate than the photogrammetric method.

Another possible measurement method is to combine both systems by using photogrammetry to coordinate acquisition in corridors and the direct method for the remaining elements. However, this requires more investment in equipment and more inconvenient transportation.

As a result of this study the company involved with the project is replacing the direct measurement method for the method based on close range photogrammetry.

## Figures and Tables

**Table 1.** Absolute and relative differences of the perimeters (mm) and areas (cm^2^) in pieces of boat decks measured by both methods.

	**Area (cm^2^)**	**Perimeter (mm)**

**Corridor R49**	336.02 (1.14%)	241.38 (0.98%)
**Cockpit R49**	44 (0.07%)	278 (2.07%)
**Cockpit R44**	149 (0.25%)	24 (0.18%)
**Cockpit R41**	204 (0.37%)	60 (0.45%)

**Table 2.** Comparison of time consumption (in minutes) for the measurement of pieces of boat decks for both of the methods used.

	**Rodman 49**				**Rodman 44**		**Rodman 41**	

	**Corridor**		**Cockpit**		**Cockpit**		**Cockpit**	

	**Phot.**	**Dir. met.**	**Phot.**	**Dir. met.**	**Phot.**	**Dir. met.**	**Phot.**	**Dir. met.**

**Acquisition**	60’	105’	50’	30’	50’	30’	45’	30’
**Processing**	75’	130’	68’	100’	65’	100’	65’	94’
